# Sustainable Hybrid Lightweight Aggregate Concrete Using Recycled Expanded Polystyrene

**DOI:** 10.3390/ma17102368

**Published:** 2024-05-15

**Authors:** D. González-Betancur, Ary A. Hoyos-Montilla, Jorge I. Tobón

**Affiliations:** 1 Materials Research Group, Construction School, Faculty of Architecture, Universidad Nacional de Colombia, Medellin 050034, Antioquia, Colombia; aahoyosm@unal.edu.co; 2 Cement and Construction Materials Research Group, Materials and Minerals Department, Faculty of Mines, Universidad Nacional de Colombia, Medellin 050034, Antioquia, Colombia; jitobon@unal.edu.co

**Keywords:** lightweight aggregate concrete, hybrid cement, polymer waste, lightweight aggregate, compressive strength, X-ray diffraction, Fourier transform infrared spectroscopy

## Abstract

Global concrete production, reaching $14\times 10^{13}$ $m^{3}/$year, raises environmental concerns due to the resource-intensive nature of ordinary Portland cement (OPC) manufacturing. Simultaneously, $32.7\times 10^{9}$ kg/year of expanded polystyrene (EPS) waste poses ecological threats. This research explores the mechanical behavior of lightweight concrete (LWAC) using recycled EPS manufactured with a hybrid cement mixture (OPC and alkali-activated cement). These types of cement have been shown to improve the compressive strength of concrete, while recycled EPS significantly decreases concrete density. However, the impact of these two materials on the LWAC mechanical behavior is unclear. LWAC comprises 35% lightweight aggregates (LWA)—a combination of EPS and expanded clays (EC) — and 65% normal-weight aggregates. As a cementitious matrix, this LWAC employs 30% OPC and 70% alkaline-activated cement (AAC) based on fly ash (FA) and lime. Compressive strength tests after 28 curing days show a remarkable 48.8% improvement, surpassing the ACI 213R-03 standard requirement, which would allow this sustainable hybrid lightweight aggregate concrete to be used as structural lightweight concrete. Also obtained was a 21.5% reduction in density; this implies potential cost savings through downsizing structural elements and enhancing thermal and acoustic insulation. X-ray diffraction (XRD) and Fourier transform infrared (FTIR) spectroscopy reveal the presence of C-S-H, C-(A)-S-H, and N-A-S-H gels. However, anhydrous products in the hybrid LWAC suggest a slower reaction rate. Further investigation into activator solution dosage and curing temperature is recommended for improved mechanical performance on the 28th day of curing. This research highlights the potential for sustainable construction incorporating waste and underscores the importance of refining activation parameters for optimal performance.

## 1. Introduction

According to the Global Cement and Concrete Association (GCCA), global concrete production is around $14\times 10^{13}$ $m^{3}/$year; as a consequence of this massive utilization, cement raw materials and concrete aggregates are the most extracted mineral resources in the world [1,2]. Therefore, reducing the extraction of non-renewable source materials is essential to preserve the natural ecosystems for future generations [3,4]. The extensive use of ordinary Portland cement (OPC) as the main binder in concrete production raises environmental concerns due to its resource- and energy-intensive manufacturing process [5], emitting 0.85–0.92 tonC$O_{2}/$tonOPC [4,6]. The accumulation of greenhouse gases, including carbon dioxide, in the atmosphere leads to climate change, melting glaciers, rising sea levels, extreme weather events, wildfires, acid rain, and disruption of natural habitats [7]. Consequently, cleaner production practices in the construction sector are imperative to mitigate the environmental impact.

On the other hand, polymers are used in almost all areas of our daily lives. They have many applications in different economic sectors, and their production has grown exponentially [8]. By the year 2050, their production is estimated to reach $33\times 10^{15}$ kg/year [9,10]. In particular, expanded polystyrene (EPS) is used as packaging or thermal insulation, generating a high volume of this plastic waste [11,12]. Around $32.7\times 10^{9}$ kg/year of EPS are generated globally [13]. From different industrial and domestic products, EPS particles are released and often fragmented into smaller ones, which are known as microplastics. EPS microplastics contain a styrene monomer, which is a carcinogen and may pose a serious threat to aquatic organisms because they may be fallaciously ingested along with food [14,15]. EPS is frequently detected with sizes of <5 mm in the marine environment [9,16].

As a result, there is a need for correct final disposal of this waste, one method of which is to incorporate it into the construction materials manufactured. Studies have shown that plastic could be used in concrete, and using recycled polymers as a replacement of natural aggregates has been explored to produce lightweight aggregate concrete (LWAC). This has been shown to increase the thermal resistivity of the envelope components [17,18,19].

### 1.1. Lightweight Aggregate Types to Manufacture LWAC

According to previous research, it is possible to state that the microstructure behavior of an LWAC depends mainly on the nature of the aggregate. Vargas et al. [20] analyzed the microstructure and thickness of the interfacial transition zone (ITZ) in concrete made with OPC and two types of lightweight aggregates (LWA)—pumice and expended clays—to determine the properties’ influence on the concrete. By characterizing the ITZ in LWAC with pumice and expanded clays, it was concluded that the densification of the ITZ, which refers to its low porosity and higher amount of hydrated phases, could be attributed to the physical and chemical properties of the lightweight aggregate structure, such as surface porosity, chemical composition, and degree of crystallinity of the aggregate. However, the LWA, due to its porous characteristics, has a lower stiffness, and, when subjected to a load, it suddenly breaks, hence the fragility observed in this type of material [20,21].

On the other hand, Brooks et al. [22] used different LWA types from waste. This study reports that, regarding the morphology of polymer-lightened concrete, EPS beads act mainly as weak points in the mortar, promoting the initiation and propagation of cracks in the concrete structure. A similar situation occurs for thermoplastic microspheres (ETM), although the particles are smaller than EPS. Therefore, as the fraction of these aggregates increases, the strength of both samples decreases continuously.

It was reported that, depending on the LWA density, the LWA is usually less resistant and stiffer than the cementitious matrix. Therefore, the LWA is decisive for the strength of the LWAC structure [23]. Angelin et al. [24] manufactured self-compacting LWACs using partial substitutions of the natural aggregate by two types of lightweight aggregates: tire rubber waste (with a maximum size of 9.5 mm and substitutions of 5%, 10%, and 15%) and expanded clays (maximum sizes of 4.8 and 9.5 mm). The microstructure of the resulting material was analyzed, and, in the case of expanded clays, the mechanical interlocking phenomenon between the aggregate and the cementitious matrix is observed, increasing the mechanical strength and durability of the concrete [20,25,26]. In the case of the waste tire rubber, no interlocking was observed between the paste and the tire rubber. Consequently, a zone of weakness occurs between the paste and the aggregate surface. Additionally, lower amounts of calcium silicate hydrate gel (C-S-H) and higher amounts of calcium hydroxide (CH) are reported, indicating a decrease in the mechanical strength of the resulting material [25].

Given the production of more significant amounts of CH in these materials, it is feasible to use hybrid cementitious materials that could achieve excellent performance by adding an alkaline source to accelerate the reaction.

### 1.2. Hybrid Cement to Manufacture LWAC

These types of cement are part of a line of research in which a cementitious material is formed from the mixture of two or more cement types [27], one of which is OPC and fly-ash-based alkaline-activated cement (AAFAC). With AAFAC, a mechanically resistant product is obtained with the reaction between solid material composed of aluminosilicates—such as fly ash (FA)—with a solution of an alkaline hydroxide—such as sodium hydroxide (SH) and sodium silicate (SS)—in this process; there is a total or partial transformation of the reactive phase, present in the aluminosilicate, to a cementitious and compact structure. Using this cementitious material has been a new alternative from the microstructural point of view for LWAC.

The study and development of hybrid cement have increased due to the possibility of being a variant to OPC since it presents the same or better properties [28]. In the case of FA, its reactivity in hybrid alkaline-activated cement is much faster and depends on the reaction conditions, particle shape, mineralogy, and particle size. In general, FA reactivity increases with increasing glassy phase content, particle size reduction, and the activator’s increasing alkalinity [29,30]. Moreover, the microstructure formed in the alkaline-activated part from a hybrid concrete has totally different features compared to that in OPC concrete. A few studies have discussed the microstructure and properties of ITZ in alkaline-activated fly ash concrete (AAFAC) and alkaline-activated slag concrete (AASC) [5,31,32,33].

Those studies found that, in AAFAC, there is no apparent weak ITZ near the aggregates due to the formation of sodium aluminosilicate gels (N-A-S-H gels) rather than CH crystals in this region, as is the case in the OPC cementitious matrix [34]. The N-A-S-H gels are the major binding phase in AAFAC, which would promote interparticle bonding and macroscopic strength in the ITZ. Additionally, the existence of soluble silicates in the initial alkaline solution would also effectively improve the interfacial bonding between aggregates and pastes in AAFAC [34]. In AASC, it was observed that the ITZ between aggregates and the paste matrix is condensed and uniform, which is attributed to the refinement of the pore structure as a result of the filling of reaction products. This is because this zone is mainly composed of N-(C)-A-S-H or C-A-S-H gels with lower Ca/Si ratios rather than the expansive (Al-free) gels [35].

In recent years, concrete manufactured from alkali-activated fly ash with an additional source of calcium as a hybrid cement system has attracted increasing attention because of its potential to provide a good synergy between mechanical properties and durability under ambient curing condition, which could not be achieved by the sole alkaline-activated concrete, e.g., AAFAC and AASC concrete [5,32,34]. García-Lodeiro et al. [29] analyzed hybrid cement activated with solutions of different alkalinity. They found that, although the type of alkaline activator affected the reaction kinetics and the formation of secondary reaction products, it did not seem to affect the nature of the main cementitious gels formed. In a hybrid cement produced by mixing OPC and an AAFAC, it is clear that the main reaction products of each coexist, i.e., hydrated calcium silicate (C-S-H) in the OPC and hydrated sodium aluminosilicate (N-A-S-H) in the AAFAC.

The high strength and low permeability of LWAC using an alternative cementitious matrix would allow obtaining a low-density concrete or mortar incorporating LWA without compromising its mechanical properties and durability. Several research studies have produced LWAC using alkali-activated cement (AAC). Posi et al. [36] evaluated the properties of geopolymer concrete using recycled LWA from construction and demolition waste. Huiskes et al. [37] produced lightweight geopolymer concrete using industrial biowaste as a precursor and polyethylene terephthalate (PET) particles as lightweight aggregates. The chemical composition and physical characteristics of the LWA significantly influence the resulting material properties. For example, Wongsa et al. [38] demonstrated that geopolymer concrete lightened with brick waste has an adequate structural behavior while concrete with the same characteristics lightened with pumice performs more appropriately in manufacturing blocks.

However, LWAC production with hybrid cement has not been reported. This has great potential since hybrid cement is the most expeditious way to achieve OPC substitution, and it has already been demonstrated that LWAC could be produced with good performance with each OPC and AAC cement separately. This research explores the mechanical behavior of lightweight concrete (LWAC) using recycled EPS manufactured with a hybrid cement mixture (OPC and alkali-activated cement). These types of cement have been shown to improve the compressive strength of concrete, while recycled EPS significantly decreases concrete density. However, the impact of these two materials on the LWAC mechanical behavior is unclear. This research paper aims to determine the effect of a hybrid cementitious matrix (composed of OPC and an AAC based on FA and lime) on the compressive strength of an LWAC.

## 2. Materials and Methods

### 2.1. Raw Materials

The characterization of the raw materials was conducted employing different instrumental techniques to identify the physical, mineralogical, and chemical properties of the materials to be worked with.

#### 2.1.1. Aggregates

This research used a mix of 35% lightweight aggregates (LWA) and 65% normal-weight aggregates (NWA) [39]. Expanded clays (EC) with a maximum particle size of 9.53 mm were used. Expanded polystyrene wastes (EPS) were used as polymer LWA, obtained from a local recycling plant and transformed by mechanical crushing to reach a maximum size of 4.75 mm, as reported by previous studies, to obtain the highest compressive strength [22,40]. Normal-weight aggregates (NWA) with a maximum particle size of 9.53 mm were obtained from a local construction materials manufacturing company. The aggregate particle size distribution was obtained by sieving the different aggregates according to ASTM C [41] 136:2014. To reduce the incidence of aggregate particle size in the microstructure of the samples, Fuller–Thompson methodology was used to standardize the aggregate, considering Equation (1), where *p* is the percentage by weight passing through a sieve, d is the sieve size, and D is the maximum aggregate size. For this research, D=9.53 mm. The ASTM C 330:2017 was used to determine aggregate density. Humidity and absorption of the aggregates were determined according to Gómez-Cano et al. [41,42] procedure. These results are presented in Table 1.

(1) $$p=100\times \sqrt{\frac{d}{D}}$$

#### 2.1.2. Cementitious Materials

**Figure 1:**
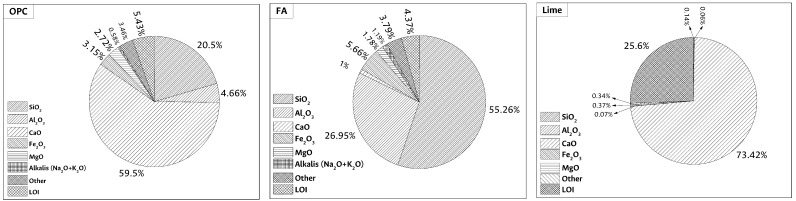
Chemical compositions of OPC, FA, and lime. Determined by XRF. Percentages of oxides by mass.

This research used two cementitious material types to fabricates the LWAC, 30% Portland cement (OPC) and 70% alkaline-activated cement (AAC) [43,44]. The 70% AAC is composed of 65% fly ash (FA) and 5% calcium hydroxide (lime) [45,46]. BET test was used to determine the specific surface area (SSA) of the materials according to ASTM C 1069. For OPC, FA, and lime, the SSA corresponds to 3270 $m^{2}/$kg, 2160 $m^{2}/$kg, and 2143 $m^{2}/$kg, respectively [39]. The chemical characterization of raw materials was performed through X-ray fluorescence (XRF) using Panalytical equipment; model Axios, through quantitative analysis over pearl per X-ray fluorescence per dispersive wavelength. The results are presented in Figure 1. The densities of OPC, FA, and lime were 3270 kg/$m^{3}$, 2160 kg/$m^{3}$, and 2143 kg/$m^{3}$ according to ASTM C188:2017. The raw materials mineralogical analysis was performed by X-ray diffraction (XRD) with the parameters described in Section 2.3.1. The amount of unburned ash was also determined based on ASTM D 3174:2018.

The glassy phase content of FA was determined according to Ibáñez et al. [47] by attacking it with 1% HF for six hours. The reactive silica content for FA was determined from the UNE 80225:2012 standard. The sample was subjected to treatment with hydrochloric acid and potassium hydroxide solution; the liquids filtered to determine the glassy phase content in the FA were analyzed by atomic spectroscopy (ICP) to determine the elemental composition of the dissolved ions and to establish the amount of reactive alumina. Data recording was performed with a plasma flow rate of 15.00 L/min, a nebulizer flow rate of 0.85 L/min, a reading time of 5 s, and a torch height of 12 mm. The detection limits of the equipment for Al and Si atoms are 0.018 and 0.069 ppm, respectively. The amount of unburned ash was also determined based on ASTM D 3174:2018.

According to ASTM C 618:2019, FA can be classified as type F [48,49]. This FA type has an acidic composition since it contains mainly silica and a small amount of calcium, as shown in Figure 1. The FA presented a reactive silica percentage of 42.20%, a glassy phase content of 65.71%, determined by attacking it with 1% HF for six hours according to Ibáñez et al. [47]. The amount of reactive alumina was 22.54%. The OPC used corresponds to a high early strength type according ASTM C 1157:2017. Finally, sodium hydroxide (SH) was used as Caustic Soda Flakes with a purity of 99%. The sodium silicate (SS) available for this research has a ${\mathrm{Na}}_{2}$O/Si$O_{2}$ ratio of 1:3, with 8.4% ${\mathrm{Na}}_{2}$O and 27.8% Si$O_{2}$ and density of 1366 kg/$m^{3}$, and the mix water is potable properly demineralized [29,31,44,45,46,50]. The industrial lime with 95.6% of calcium hydroxide and 4% calcium carbonate content (measured by XRD using the Rietveld method. $R_{wp}=12.5\%$) was employed.

**Figure 2:**
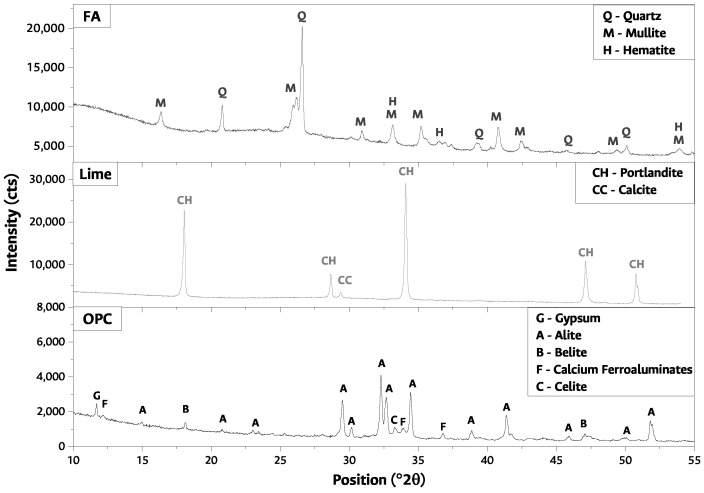
Fly ash, lime, and ordinary Portland cement X-ray diffraction.

Figure 2 shows the qualitative mineralogical composition for FA and lime. The presence of a low degree of crystallinity phase between 20 and $40^{\circ }2\theta$ and three crystalline phases, mullite (M), quartz (Q), and hematite (H), is observed for FA. Portlandite (CH) and calcium carbonate (T) are noticed in lime [44,51]. The mineralogical composition of the OPC used in this research presents a more amorphous composition than the other raw materials due to the halo observed between $30^{\circ }$ and $35^{\circ }2\theta$. This material presents the typical mineralogy of an OPC for structural use, with high early strength, due to the intensity and repetitiveness of the alite peaks in the diffractogram. In addition, it shows calcium ferroaluminates, and some belite and gypsum peaks.

### 2.2. Concrete Fabrication and Mechanical Test

The LWAC mixtures were prepared considering solids FA, OPC, and lime. The liquid was formed by deionized water, SS, and SH [52]. The lime participates by weight substitution in the dosage of FA used in the alkali-activated cement and both replaced cement.

All solid components were homogenized in a ball mill. Subsequently, the liquid part was prepared by dissolving caustic soda in deionized water using a magnetic stirrer until the solution reached room temperature. A 0.49 liquid-by-solid ratio (L/S) was used for all the mixes evaluated. The amount of aggregate by absolute volume was also obtained. Once the cement paste was prepared, the corresponding aggregate volume was added, and the mixture was homogenized and compacted in 5×5×5 cubic molds. The mix design methodology proposed by Pavithra et al. [52] was used to calculate the mixing ratios. Mix proportions for mixes fabricated in this study are presented in Table 2.

Three specimens were manufactured per type of mixture. The samples were cured in an airtight container at an average relative humidity of 98%, exposed to 45 °C for three days [44,51,53]. Once this time was over, samples were taken to a curing chamber, preserving room temperature and humidity 98% until 28 days of curing. After reaching the age of curing, the compressive strength test to the corresponding samples was conducted in compliance with ASTM C 109M:2016, and the average compressive strength was obtained for each kind of mix.

### 2.3. Cementitious Matrix Analysis

#### 2.3.1. X-ray Diffraction Analysis (XRD)

Mineralogical identification of the hydration products of the cementitious material was completed using reference Panalytical XPert PRO MPD in an interval °2θ between $5^{\circ }$ and $70^{\circ }$, with a step size $0.026^{\circ }$ and an average time by step 60 s. A copper anode with Kα=1.5406 $\overset{˚}{A}$ was used.

#### 2.3.2. Fourier Transform Infrared Spectroscopy (FTIR)

The analysis of the functional groups of the cement pastes was performed with Fourier transform infrared spectroscopy (FTIR) in an Advantage FTIR 8400 SHIMADZU. The number of scans was 64, with a resolution of 4 ${\mathrm{cm}}^{-1}$. Tablets were prepared using 1 mg of sample (with a particle size of approximately 75μm) mixed with 100 mg of dry KBr, the recording was performed mid-infrared, and logging was completed between 4000 ${\mathrm{cm}}^{-1}$ and 400 ${\mathrm{cm}}^{-1}$.

Additionally, to facilitate the identification of overlapping bands and to extract valuable information from the hybrid cement FTIR spectroscopy main band, a deconvolution was performed using a Gaussian fit with fit R2=0.99782.

## 3. Results and Discussion

### 3.1. Compressive Strength

**Figure 3:**
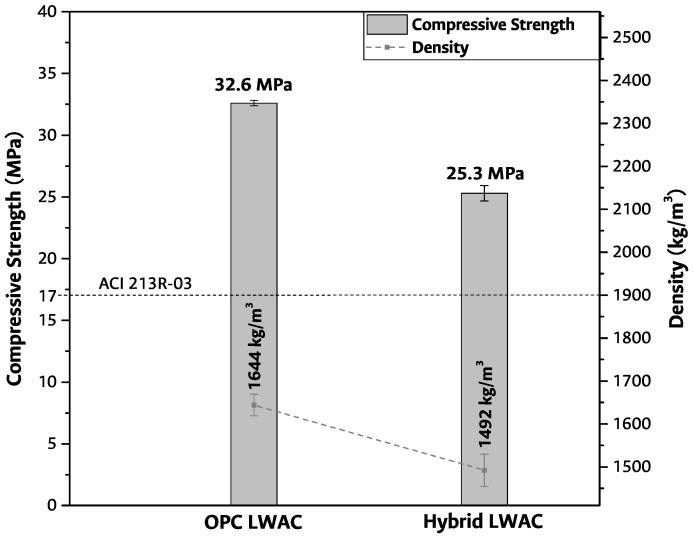
Compressive strength and density for 28 days of curing.

The hybrid LWAC manufactured was compared to an LWAC using OPC as a cementitious matrix by compressive strength. In Figure 3, the compressive strength and density of the OPC LWAC and hybrid LWAC for 28 days of curing are shown. Moreover, these results were compared with the ACI 213R-03 minimum requirements for compressive strength (minimum 17 MPa) and density (maximum 1900 kg/$m^{3}$) for LWAC.

A significant reduction in density is observed when comparing the density obtained for the lightweight concretes (OPC LWAC and hybrid LWAC) and the maximum density established in the standard. Considering that OPC LWAC and hybrid LWAC contain the same type of aggregate, in terms of material density, there is a 9% reduction as the cementitious matrix is modified. This density reduction can be explained by the hybrid LWAC’s materials having densities much lower than those of the OPC LWAC, as was presented in Section 2.1.2. When the results are compared with the maximum density requirements in ACI 213R-03, reductions of 13.5% and 21.5% for OPC LWAC and hybrid LWAC in density were obtained. According to ACI 213R-03, the minimum compressive strength at 28 days of curing for a structural LWAC is 17 MPa; the hybrid LWAC obtained in this research is above the minimum requirement by up to 8.3 MPa. This implies a 48.8% improvement in mechanical behavior for the hybrid LWAC.

At this point, it is essential to highlight that the hybrid LWAC obtained in this research could be used to design a concrete structure, demonstrating that it is possible to manufacture an LWAC in compliance with the minimum requirements of the ACI 213R-03 standard for structural construction materials that incorporates waste both in its aggregates and in its cementitious matrix. Additionally, this proposes an alternative use for waste with no commercial value for the construction industry at present, such as expanded polystyrene wastes. In that case, reducing the cross-section of the structural elements in a building construction would be possible, and, furthermore, its thermal and acoustic insulation could be increased, as reported in the literature [17,18,19], thus decreasing the dead loads directly related to its own weight. It could finally be translated into savings in the total cost of the construction work.

It is important to note that, with a hybrid cement (that uses an AAC and OPC), the mechanical properties mainly depend on the structure and chemical composition of the reaction products, the ions concentration, and the raw material reactivity [31]. On the other hand, The hydroxyl (${\mathrm{OH}}^{-}$), catalyzer (${\mathrm{Na}}^{+}$) content, and curing temperature control the AAC reaction rate. A higher amount of free ions, a high amount of catalyzer, and a temperature of curing that accelerates the FA reaction improve the formation rate of the main hydration product—responsible for the compressive strength in concretes—C-(A)-S-H gel in the AAC fraction [27]. In the case of the hybrid LWAC studied in this research, the reduction in compressive strength with respect to OPC LWAC might be due to a difference in the reaction rate of the two cementitious matrices at 28 days of curing. This implies that the amount of activating solution or the curing temperature were insufficient to achieve a compressive strength equal to that of the OPC LWAC sample.

In an attempt to understand the compressive strength development of the samples, they were analyzed by XRD and FTIR techniques to identify the hydration products formed and compare the reaction grade for OPC and hybrid cement, which could confirm the difference in compressive strength and will be discussed in the following section.

### 3.2. Cementitious Matrix Analysis

#### 3.2.1. X-ray Diffraction Analysis

**Figure 4:**
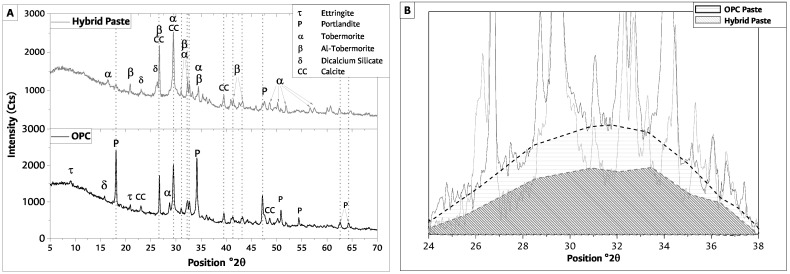
(**A**) X-ray diffraction and (**B**) variation in the short-range crystallinity between samples at 28 days.

The X-ray diffractograms for OPC and hybrid LWAC at 28 days of curing are reported in Figure 4A. Additionally, an ampliation of XRD analysis in this study, presented in Figure 4B, is undertaken to elucidate the intricate structural changes occurring within hybrid cement paste compared to OPC paste.

OPC analysis confirmed that the cement hydrated normally due to the presence of C-S-H gel. This compound presents a crystallographic order to XRD [54,55]. Secondary hydration products such as portlandite (Ca(OH)_2_) and ettringite were also observed in the hydrated cement, as expected. The XRD analysis for hybrid cement indicates the C-A-S-H and C-S-H prevail under the N-A-S-H gels. This behavior was reported previously due to the increment of calcium source in cement [31,46].

Additionally, as was approached by Hoyos-Montilla et al. [45,46] and considering that the CH particle size is, on average, six times smaller than that of FA, CH could form a film on the FA surface during the mixing process of the solids. When the solid part comes in contact with the alkaline activator solution, silicon diffuses first to sites with higher sodium presence and in a smaller proportion to sites with calcium presence. In contrast, aluminum diffuses more slowly than silicon, but the presence of calcium stimulates its displacement towards the CH boundary. Because the lattice-forming elements must pass through the CH layer, the first gel formed is the C-(A)-S-H gel around the dissolving FA [44,45,46]. The bridging units of the C-(A)-S-H gel act as starting seeds for forming the N(C)-A-S-H gel, which develops between the existing spaces of the gel layers covering the dissolving FA. As the concentration of aluminum increases toward the FA, it is possible that, between the unreacted FA and the CH layer, there is a primary gel formed after dissolution, which is rich in aluminum. A structural reorganization directly affects the compressive strength of the cementitious material [44,45,46,50].

Carbonates, essentially CaC$O_{3}$, were observed in all the samples. The presence of calcium carbonate had a dual explanation. It was present in the anhydrous lime (used for hybrid cement) and generated in the hydrated pastes as a result of portlandite carbonation by atmospheric ${\mathrm{CO}}_{2}$ [55]. It translated into a decline in the intensity of its diffraction lines on the 28th day pattern and a rise in the lines associated with carbonates. In addition to carbonating, portlandite may react with the silica and alumina in the FA to generate C-(A)-S-H-gel-like products (pozzolanic reaction) [55].

Figure 4B presents a variation in the short-range crystallinity of the hybrid cement paste concerning the OPC cement paste. These changes are related to forming new reaction products, mainly an alkali aluminosilicate gel with a short-range structural order, such as C-A-S-H [51]. The variation in the intensity of the peak is related to the dissolution of the reactive material of the raw materials that compose the hybrid cement as a function of the amount of activator [56]. Calcium presence modifies the hydrate product’s glass structure [29,46,50] and contributes to a higher presence of C-(A)-S-H gel products. A mixture in the presence of CH forms crystalline or semi-crystalline phases of two types, the first of zeolitic character (Ca_49.10_Si_96_Al_96_O_384_) and the second of hydrated calcium silicates (Ca_1.5_SiO_3.5_xH_2_O) [51,57].

In Figure 4B, differences in their intensities are observed. This could be due to a lower formation of gels in the hybrid cement paste sample and could be related to the reaction rate of the two cementitious matrices at 28 days of curing, as reported by Suh et al. [58]. As stated above, the reduction in mechanical behavior of a hybrid LWAC depends on the materials’ reaction rate at the evaluation time. The hydroxyl (${\mathrm{OH}}^{-}$) and catalyzer (${\mathrm{Na}}^{+}$) content in the activator solution, and curing temperature, control the hybrid cement reaction rate, implying that the amount of activating solution or the curing temperature were insufficient to achieve mechanical behavior equal to that of the OPC LWAC. This could explain the compressive strength difference of around 7.3 MPa between the LWAC hybrid and LWAC OPC samples. However, there is a potential for reaction at advanced curing ages, so it is recommended to adjust the use of activating solution and curing temperature to improve mechanical behavior at 28 days of curing.

#### 3.2.2. Fourier Transform Infrared (FTIR) Spectroscopy Analysis

**Figure 5:**
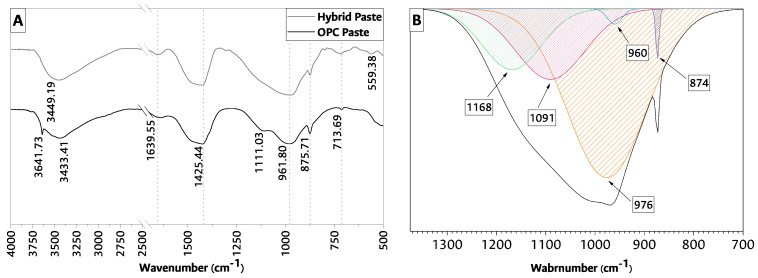
(**A**) FTIR for studying samples and (**B**) main band deconvolution for the hybrid sample at 28 days.

The FTIR present in Figure 5A for paste samples at 28 days of curing represent a powerful analytical technique that provides insights into the molecular composition, structure, and behavior of chemical compounds. Additionally, deconvoluting curves in FTIR spectroscopy is an essential data processing step aimed at untangling these complexities and extracting valuable information; by isolating the principal band in the spectrum and characterizing their positions and intensities, as deconvolution facilitates peak identification, the principal bands in the hybrid FTIR were deconvoluted in Figure 5B.

From Figure 5A, it can be seen that the spectra obtained for the two samples have bands in common: the broad bands around 3400 ${\mathrm{cm}}^{-1}$ and 1640 ${\mathrm{cm}}^{-1}$ are assigned to the stretching and curvature vibrations of ^−^OH in water, respectively. When OPC and hybrid cement are compared, the bands corresponding to the release of O-H groups present in the hydration products and their reaction with the other mixture elements cause a shift of the wavenumber towards higher values, which could explain the slight difference between the bands 3433 ${\mathrm{cm}}^{-1}$ and 3439 ${\mathrm{cm}}^{-1}$ from the OPC and hybrid pastes shown in Figure 5A. These changes are due to the presence of Ca-O-H units and the capture of mainly silicon and aluminum atoms by hydroxyl groups to form Si(Al)-O-H structures [57]. These structures and units increase in time due to the formation of new products.

Furthermore, the band 3641.73 ${\mathrm{cm}}^{-1}$ is particularly associated with ${\mathrm{OH}}^{-}$ stretching vibrations in Ca(OH)_2_ [55]. The bands centered at 1426 ${\mathrm{cm}}^{-1}$ and 876 c$m^{-1}$ indicate the asymmetric deformation vibration and the C$O_{3}^{2-}$ wiggle vibration characteristic of calcite. The band around 713 ${\mathrm{cm}}^{-1}$ represents the main calcite band [55,59]. This phenomenon is caused due to the presence of calcium carbonate in the CH added to the mixtures and later by the formation of sodium carbonates during the alkaline activation, causing the area to increase [51]. This result is consistent with the XRD findings (Figure 4).

The main band around 960 ${\mathrm{cm}}^{-1}$ is associated with asymmetric stretching vibrations of Si-O bonds and bands located at lower wavenumbers assigned to deformation vibrations (Si-O-Si/Si-O-Al) [60]. Therefore, these spectra indicate structural features similar to C-A-S-H gel structures.

Particularly, the band around 560 ${\mathrm{cm}}^{-1}$, present in the hybrid sample, is attributed to the tetrahedral stretching bands of aluminum. The bands in the 690 ${\mathrm{cm}}^{-1}$ to 440 ${\mathrm{cm}}^{-1}$ range are associated with the bending vibrations generated by the Si-O-Si/Si-O-Al bonds, some characteristic bands of N-A-S-H gels. A band was likewise observed in the 800 ${\mathrm{cm}}^{-1}$ zone, associated with vibrations in the tetrahedra that form the so-called secondary building units (SBU) and fragments of the aluminosilicate network. All this suggests that these gels generally had more intensely polymerized and very likely three-dimensional structures [60]. Bands are present in OPC in 1100 ${\mathrm{cm}}^{-1}$ to 1165 ${\mathrm{cm}}^{-1}$, corresponding to ${\mathrm{SO}}_{4}^{-2}$ in sulfates. This region presents a range of bands that may overlap due to the polymerization of ${\mathrm{SO}}_{4}^{-2}$ and the corresponding vibration [59].

Figure 5B revealed two key components: one at 976 ${\mathrm{cm}}^{-1}$ (a less prominent band at 960 ${\mathrm{cm}}^{-1}$) and the other at 1090 ${\mathrm{cm}}^{-1}$, corresponding to C-(A)-S-H and N-A-S-H, respectively. The band at about 976 ${\mathrm{cm}}^{-1}$ represents the Si-O vibrations in the C-S-H groups, and the band around 960 ${\mathrm{cm}}^{-1}$ is associated with asymmetrical stretching vibrations of Si-O bonds ($\nu_{as}$ Si-O-Si). This corroborates the presence of such a gel observed in XRD (Figure 4), indicating that the hybrid cement paste incorporated CH into its structure (as in the conventional C-S-H gel) [55,59,60]. The second key band at 1090 ${\mathrm{cm}}^{-1}$ shows the already well-recognized substantial shift in position and shape of the $\nu_{as}$ Si-O absorption band associated with N-A-S-H gel [61,62]. In other words, this deconvoluted signal closely resembled the bands of the C-(A)-S-H gel and poorly reached the peaks of the N-A-S-H gel. This implies that, in this case, the synthesized mixture comprised two fractions, one with a calcium-rich gel and one with a silica-rich gel.The band centered at 874 c$m^{-1}$ indicates the asymmetric wagging vibration of C$O_{3}^{2-}$ in calcite.

It has been previously shown in the literature [46,51] that the bands corresponding to the Si−O groups are found in a shoulder around the 1170 ${\mathrm{cm}}^{-1}$ band. This shoulder tends to disappear as the curing age of the AAC advances, resulting in compounds from alkaline activation [46]. In the case of Figure 5B, this shoulder is still present, indicating that a fraction of the FA remains anhydrous at the 28th day of curing, which promotes failure surfaces in the material, reducing the compressive strength as a consequence. However, there is a potential for reaction at advanced curing ages, so it is recommended to adjust the use of activating solution and curing temperature to improve mechanical behavior at 28 days of curing.

The investigation regarding hybrid LWAC revealed promising results, with the compressive strength surpassing the minimum requirement set by ACI 213R-03 by up to 48.8%. Simultaneously, the hybrid LWAC exhibited a 13.5% reduction in density compared to the standard, suggesting the potential for reducing the cross-section of structural elements and also enhancing thermal and acoustic insulation in building construction. The analysis of the hybrid sample using XRD and deconvoluted FTIR identified the presence of key components—C-S-H and C-(A)-S-H groups—indicating the incorporation of CH into its structure. This incorporation led to a higher cross-linking chain reaction product. However, the study also noted the presence of anhydrous products in the hybrid LWAC, hinting at potential issues with the activator solution amount or curing temperature. This observation suggests the need for further investigation and adjustments to ensure optimal outcomes, possibly by addressing factors like activator solution usage to enhance the dissolution rate of FA and prevent compromise to compressive strength.

## 4. Conclusions

The present study investigated the effect of replacing cement and aggregates in conventional concrete with a hybrid cement and LWA on the compressive strength regarding mechanical behavior and phase compositions. Under the conditions and scope of this experimental study, the main conclusions drawn are summarized below:

- This study demonstrated the feasibility of producing lightweight concrete in compliance with the standard outlined in ACI 213R-03 for structural lightweight concretes, exceeding the minimum compressive strength by 48.8% and reducing the density by up to 21.5%. The innovation lies in incorporating waste materials into both the aggregates and cementitious matrix. A novel application proposes utilizing low-value waste, like expanded polystyrene, currently overlooked by the construction industry. This could not only enable a reduction in structural element cross-sections but also could enhance thermal and acoustic insulation in building construction. Consequently, a decrease in dead loads, directly linked to the material’s weight, offers potential cost savings in overall construction expenses.
- The XRD and the deconvoluted FTIR analysis identified partially hydrated FA in the hybrid lightweight aggregate concrete (LWAC), which promotes failure surfaces in the material, reducing the compressive strength in consequence. However, it is crucial to recognize the potential for delayed reactions at higher ages. This potential underscores the importance of thorough exploration and optimization of curing parameters, offering a pathway to unlock the full potential of this material in bolstering its mechanical properties over time. Further investigation and adjustments to the activator solution usage might be recommended to ensure optimal outcomes.
- This research revealed the presence of two key components in the hybrid sample C-S-H and C-(A)-S-H groups, with a few bands corresponding to the N-A-S-H gel. This indicates that the hybrid sample incorporated CH into its structure—as in the OPC hydration products—leading to a higher cross-linking chain reaction product.

## Figures and Tables

**Table 1:** Aggregate properties.

Aggregate	ρ ^1^ (g/c$m^{3}$)	Absorption (%)	SSA ^2^ ($m^{2}$/g)
EPS	0.01	0.1	−
EC	1.10	17.0	1.89
NWA	2.74	0.70	0.78

^1^ ρ (kg/$m^{3}$), bulk density. ^2^ SSA ($m^{2}$/kg), specific surface area.

**Table 2:** Mix proportions.

Mix Type	% Binder Composition			% Aggregate Composition			L/S	Mix Composition (g)								
	**OPC**	**FA**	**Lime**	**NWA**	**EPS**	**EC**		**OPC**	**FA**	**Lime**	**SH**	**SS**	**Water**	**NWA**	**EPS LWA**	**EC LWA**
OPC LWAC	100	0	0	65	15	20	0.49	897	-	-	-	-	439.5	798.5	184.3	245.7
Hybrid LWAC	30	65	5	65	15	20	0.49	269.1	583.1	44.8	287.5	152.2	-	798.5	184.3	245.7

## Data Availability

Data are contained within the article.
